# Intestinal Epithelial Cells Respond to Chronic Inflammation and Dysbiosis by Synthesizing H_2_O_2_

**DOI:** 10.3389/fphys.2019.01484

**Published:** 2019-12-12

**Authors:** Juan F. Burgueño, Julia Fritsch, Ana M. Santander, Nivis Brito, Irina Fernández, Judith Pignac-Kobinger, Gregory E. Conner, Maria T. Abreu

**Affiliations:** ^1^Division of Gastroenterology, Department of Medicine, Leonard M. Miller School of Medicine, University of Miami, Miami, FL, United States; ^2^Department of Microbiology and Immunology, Leonard M. Miller School of Medicine, University of Miami, Miami, FL, United States; ^3^Department of Cell Biology, Leonard M. Miller School of Medicine, University of Miami, Miami, FL, United States

**Keywords:** colitis, intestinal inflammation, inflammatory bowel disease, microbiome, host-microbe interactions

## Abstract

The microbes in the gastrointestinal tract are separated from the host by a single layer of intestinal epithelial cells (IECs) that plays pivotal roles in maintaining homeostasis by absorbing nutrients and providing a physical and immunological barrier to potential pathogens. Preservation of homeostasis requires the crosstalk between the epithelium and the microbial environment. One epithelial-driven innate immune mechanism that participates in host-microbe communication involves the release of reactive oxygen species (ROS), such as hydrogen peroxide (H_2_O_2_), toward the lumen. Phagocytes produce high amounts of ROS which is critical for microbicidal functions; the functional contribution of epithelial ROS, however, has been hindered by the lack of methodologies to reliably quantify extracellular release of ROS. Here, we used a modified Amplex Red assay to investigate the inflammatory and microbial regulation of IEC-generated H_2_O_2_ and the potential role of Duox2, a NADPH oxidase that is an important source of H_2_O_2_. We found that colonoids respond to interferon-γ and flagellin by enhancing production of H_2_O_2_ in a Duox2-mediated fashion. To extend these findings, we analyzed *ex vivo* production of H_2_O_2_ by IECs after acute and chronic inflammation, as well as after exposure to dysbiotic microbiota. While acute inflammation did not induce a significant increase in epithelial-driven H_2_O_2_, chronic inflammation caused IECs to release higher levels of H_2_O_2_. Furthermore, colonization of germ-free mice with dysbiotic microbiota from mice or patients with IBD resulted in increased H_2_O_2_ production compared with healthy controls. Collectively, these data suggest that IECs are capable of H_2_O_2_ production during chronic inflammation and dysbiotic states. Our results provide insight into luminal production of H_2_O_2_ by IECs as a read-out of innate defense by the mucosa.

## Introduction

The gut epithelial barrier consists of a single layer of IECs that separates the largest compartment of the host’s immune system from an environment with a high microbial and antigenic load (Mowat and Agace, 2014; Sender et al., 2016). This epithelial monolayer acts as a physical, chemical, and immune barrier, minimizing the contact between the host and the microbiota and therefore maintaining homeostasis. While epithelial-dependent immune functions, such as the secretion of mucus, the production of antimicrobial peptides, and the transportation of immunoglobulin A, have been widely investigated, the functions of IEC-generated ROS as signaling intermediates in host defense are still not well understood due to the lack of reliable methodologies to measure extracellular release of ROS.

Reactive oxygen species are reduced metabolites of oxygen that can oxidize other molecules and have microbicidal or signaling functions depending on their concentration in the microenvironment (Mittal et al., 2014). High levels of ROS produced by neutrophils and other phagocytes play important microbicidal functions during an oxidative burst. Under such conditions, ROS are likely to act as microbicidal agents in conjunction with MPO, which uses H_2_O_2_ to oxidize chloride forming the antimicrobial hypochlorous acid (Klebanoff et al., 2013). On the other hand, in steady-state conditions, low amounts of ROS released by non-phagocytic cells activate both host and microbial signaling pathways by altering the phosphotyrosine signaling network and inducing certain transcription factors (Holmstrom and Finkel, 2014). ROS are generated either as byproducts of mitochondrial oxidative phosphorylation or through NADPH oxidases, including Nox 1 through 5 as well as Duox 1 and 2 (Mittal et al., 2014; Aviello and Knaus, 2017). In the gut epithelium, the two major NADPH oxidases are Nox1 and Duox2 (Grasberger et al., 2015; Aviello and Knaus, 2018). Duox2 generates H_2_O_2_, whereas Nox1 produces superoxide (O_2_^–^), which can in turn be converted into H_2_O_2_ by superoxide dismutase (Dikalov and Harrison, 2014). Given its stability and diffusibility, H_2_O_2_ produced by epithelial NADPH oxidases can diffuse to the extracellular milieu and act as a messenger to communicate with neighboring host and microbial cells (Aviello and Knaus, 2017). Indeed, IEC-generated ROS participate in mucosal healing processes by inactivating diverse phosphatases that regulate cell migration (Swanson et al., 2011; Leoni et al., 2013; Moll et al., 2018). Furthermore, ROS interact with microbes, modifying bacterial proteins involved in signal transduction, virulence, motility, and invasiveness, ultimately restraining colonization of the mucosa (Botteaux et al., 2009; Corcionivoschi et al., 2012; Grasberger et al., 2013; Hayes et al., 2015).

Inflammatory bowel diseases are characterized by dysbiosis, disproportionate immune responses against the microbiota, and high levels of ROS (McKenzie et al., 1996; Gitter et al., 2001; Hussain et al., 2003; Itzkowitz and Yio, 2004; Tamboli et al., 2004; Luceri et al., 2019). Even though the high oxidative stress in IBD patients is likely due to the oxidative burst of infiltrating neutrophils, epithelial Nox1 and Duox2 are overexpressed in IBD patient biopsies (MacFie et al., 2014; Chu et al., 2017). Furthermore, Duox2 is an early upregulated gene in very early onset (VEO) IBD and loss of function mutations in both Nox1 and Duox2 have been associated with such disease (Haberman et al., 2014; Hayes et al., 2015; Parlato et al., 2017). Although it is still unclear whether the upregulation of epithelial NADPH oxidases in IBD perpetuates inflammation, some models have proposed a role for Nox1 and Duox2 in facilitating dysbiosis by providing anaerobic substrates for facultative anaerobes to bloom (Winter et al., 2013; Zhu et al., 2019). However, the fact that Duox2 is upregulated after humanization of germ-free mice with human dysbiotic microflora and prevents microbes from invading the mucosa suggests that epithelial ROS may be necessary to control dysbiosis and promote epithelial repair (Grasberger et al., 2013, 2015).

Given that ROS mediate the communication between the epithelial barrier and the mucosa-associated microbiota and have also been associated with dysbiosis and inflammation, we sought to characterize the extracellular release of H_2_O_2_ by IECs in these settings. To this end, we measured the oxidation of 10-Acetyl-3,7-dihydroxyphenoxazine (Amplex Red) into resorufin in the presence of HRP and H_2_O_2_ in IECs. This method, which is highly sensitive, allowed for real time measurement of H_2_O_2_ produced by IEC in the nanomolar range (Zhou et al., 1997). To overcome the technical difficulties posed by carboxylesterases, which are expressed in IECs and oxidize Amplex Red (AR) in an HRP-independent way, we supplemented our reaction buffers with the serine protease inhibitor PMSF, as previously described in other cell types from liver and kidney (Miwa et al., 2016). Here, we demonstrate that upon inflammatory and microbial challenge, primary IECs in culture release H_2_O_2_ to the extracellular milieu in a Duox2-mediated mechanism. Furthermore, we report that freshly isolated IECs synthesize H_2_O_2_ in response to chronic inflammation and dysbiosis. Our findings support a key role for Duox2 in mediating mucosal responses to dysbiosis.

## Materials and Methods

### Human Tissue and Stool Acquisition

The acquisition and use of human data, biopsies, and stool samples were approved by the University of Miami, Miller School of Medicine Institutional Review Board. At the time the patient samples were collected, a complete history and assessment of disease was completed (Supplementary Table 1). Colonoscopic biopsies were obtained from 6 non-inflamed IBD patients and processed the same day for organoid culture. Stool from IBD patients in remission and HS was aliquoted in an anaerobic chamber (Coy) the same day of collection and stored in −80^°^C.

### Animals

C57Bl/6 and Nox1 total knock-out (Nox1-KO; Nox1^TM1Kkr^) mice were purchased from Jackson Laboratory. Epithelial DuoxA1/A2-KO mice, which are functionally deficient in Duox1 and Duox2, were obtained by crossing the DuoxA1/A2-floxed mice generated at Dr. Kaunitz’s laboratory (UCLA) with villin-cre (Tg(Vil1-cre)997Gum) mice purchased from Jackson Laboratory. Even though these mice are double knock-out for Duox isoenzymes, they are widely accepted as a model to investigate the role of Duox2 in the colon given that the expression of Duox1 in the gut is exceedingly low (Grasberger et al., 2015; Aviello and Knaus, 2018; van der Vliet et al., 2018). Villin-TLR4 mice, which express a transgene that renders TLR4 constitutively active under the villin promoter, were generated as previously described (Shang et al., 2008). Germ-free adult C57Bl/6 mice were bred and housed at the University of Miami Gnotobiotic Facility in Class Biologically Clean flexible film isolators and transferred to Biocontainment Unit (BCU) cage system after microbial transfer. All mice were generated on a C57Bl/6 background, housed in either specific pathogen-free or germ-free conditions with a controlled temperature of 20 ± 2°C, and allowed free access to food and water, either regular or autoclaved for specific pathogen-free and germ-free mice, respectively. All experiments were performed with the approval of the Institutional Animal Care and Use Committee (IACUC) at the University of Miami (Protocols 17-196 and 18-169). The University of Miami is internationally accredited by the Association for Assessment and Accreditation of Laboratory Animal Care (AAALAC).

### Experimental Design, Colitis Induction, and Collection Samples

Mice between 8 to 16 weeks of age, of both sexes, were randomly assigned cages and then adapted for at least 5 days prior to starting experiments. In order to induce colitis, 3% of DSS (40-50 kDa; Affymetrix/USB, ThermoFisher Scientific) was added to the drinking water for either 2, 4, or 6 consecutive days and replaced every other day. To minimize variability in our determinations, all mice were euthanized on the same day (Figure 3B). The AOM-DSS model of colitis-associated cancer (CAC) was utilized in order to investigate chronic inflammation and tumorigenesis. AOM (7.4 mg/kg; Sigma-Aldrich) was injected intraperitoneally 1 week before beginning a 5-day cycle of 3% DSS. After the first cycle of DSS, the mice were allowed to recover for 2 weeks before subsequently starting a second 5-day cycle of 3% DSS. Mice were euthanized on day 56 (Figure 3E). The weight loss for each mouse was assessed daily during the DSS cycles and the first week of recovery for the CAC model as previously described to evaluate the severity of colitis. Mice losing more than 30% of initial body weight or displaying severe bloody diarrhea with lack of exploratory behavior were immediately euthanized. No animals died or met the endpoint criteria prior to the end of the study.

Mice were euthanized by cervical dislocation under isoflurane (Piramal Critical Care) anesthesia. Subsequently, the colon was removed, flushed, cut along the mesenteric border, and pinned flat on a Sylgard TM-coated Petri dish. One longitudinal section of the colon was prepared for histology by rolling it into a Swiss roll and fixing it in 4% paraformaldehyde. The rest of the colon was utilized for either IEC isolation, MPO determination, or preparation of mucosa-associated microbiota homogenates.

### Microbial Engraftment

Mucosa-associated microbiota homogenates were prepared from C57Bl/6J or villin-TLR4 mice by homogenizing flushed colons in Hank’s balanced salt solution (HBSS) using a BeadBlasterTM24 (Benchmark) in a vinyl anaerobic chamber (Coy). One mL of HBSS was used for every 50 mg of colon. To prepare the human stool samples, frozen stool was mixed with sterile HBSS (1:10 ratio of stool weight:HBSS) and filtered with a 40 μm strainer in an anaerobic chamber. GF mice were then orally gavaged with 200 uL of either the resulting mucosa-associated microbiota or stool slurry and housed in separate iso-cages depending on the donor microbiome (C57Bl/6J vs. vilin-TLR4; HS vs. IBD). After a 3-week engraftment (Figures 4A, 5A), mice were euthanized as indicated above. The colon of these mice was used for same histopathological, enzymatic, and transcriptomic determinations.

### DNA Extraction and 16S Sequencing

Total bacterial DNA was extracted, quantified, and sequenced at the University of Minnesota Genomic Center (UMGC) as previously described (Gohl et al., 2016). Briefly, DNA was extracted using PowerSoil/fecal DNA Isolation Kit (MoBio Laboratories) and the 16S-V4 region was amplified using Meta_V4_515F and Meta_V4_806R primers, and KAPAHiFidelity Hot Start Polymerase PCR. After a first amplification, the products were diluted 1:100, 5 uL of which were used for the second round of amplification. Various different combinations of forward and reverse indexing primers were used for the second PCR. For sequencing, pooled samples were denatured with NaOH, diluted to 8 pM in Illumina’s HT1 buffer, spiked with 15% PhiX, and denatured at 96°C for 2 min. A MiSeq600 cycle v3 kit was used to sequence the DNA, and Nextera adapter sequences were used for post-run trimming.

### Microbiome Data Analysis

The paired-end sequences were obtained as forward and reverse demultiplexed fastq files from the Illumina MiSeq. CosmosID bioinformatics software package (CosmosID Inc.) was used to identify bacterial species, quantify the relative abundance, calculate alpha diversity, and generate a principal component analysis. Briefly, CosmosID pipeline uses a high-performance data-mining k-mer algorithm that rapidly disambiguates millions of short sequencing reads into the discrete genomes engendering the particular sequences. This pipeline has two separable comparators that include a pre-computation phase that matches a k-mer to a uniquely identified reference database and a per-sample computation that statistically scores the entire read to verify the identification and to avoid false positive identifications. For the pre-computation phase, the input is microbial genome databases, and the output is phylogeny trees and variable length k-mer fingerprints (biomarkers) that uniquely identify nodes generating branches and leaves of the tree. While the second per-sample computation phase searches 100s of millions of short sequence reads or contigs from a draft assembly against fingerprint sets. Finally, to obtain fine-grain taxonomic and relative abundance estimates for the microbial datasets, the resulting statistics are analyzed.

### Isolation of IECs and Colonoid Preparation

In order to isolate IECs, the murine colonic sections and human biopsies were shaken at 150 rpm for 45 min at room temperature in a chelation buffer prepared with 10 mM EDTA in HBSS. The residual EDTA was then gently washed with HBSS, and the crypts were released by gentle agitation for 30 min. The remaining longitudinal tissue was removed and snap frozen for future MPO experiments. The released crypts were either used to generate colonoids, utilized for the direct measurement of H_2_O_2_, or lysed in Trizol (ThermoFisher) for qPCR. To generate colonoids, the IECs were pelleted and resuspended in ice-cold Cultrex reduced growth factor basement membrane, type R1 (R&D systems). For 2 days, mouse colonoids were grown in 50% conditioned medium containing wnt3a, R-spondin-3, noggin, and 20% fetal bovine serum (WRNC medium) (Miyoshi and Stappenbeck, 2013) supplemented with 5 μM Chir99021 (Cayman Chemical), 2.5 μM Thiazovivin (Cayman Chemical), and 100 μg/mL Primocin (Invivogen). Human colonoids were grown in a mixture of Human Intesticult Organoid Growth Medium (STEMCELL Technologies) and WRNC medium (1:3 ratio) and supplemented with 5 μM Chir99021, 2.5 μM Thiazovivin, 0.5 μM A83-01 (Cayman Chemical), 10 μM SB202190 (Cayman Chemical), 100 μg/mL Primocin, and 1x gentamicin-amphotericin B (ThermoFisher). After 2 days, colonoids were switched to a growth factor-restricted, less reactive medium containing DMEM/F12, 10% R-spondin-2 (Bell et al., 2008) and 10% noggin-conditioned media (Heijmans et al., 2014), 10% fetal bovine serum, mouse epidermal growth factor (50 ng/mL; Life Technologies), Primocin (100 μg/mL) and Chir00921 (5 μM) (RENC medium). Colonoids stimulated on day 6 for 24 h with or without 100 ng/mL interferon (IFN)γ (Biolegend) or 100 ng/mL ultrapure *Salmonella typhimurium* flagellin (Invivogen) in RENC were utilized for H_2_O_2_ measurement.

### Real-Time Measurement of Hydrogen Peroxide Production

Intestinal epithelial cells or colonoids seeded in 96 well plates were incubated in Dulbecco’s PBS (DPBS) solution containing Ca2^+^, Mg2^+^, 0.1 U/mL HRP (Sigma Aldrich), 30 μM 10-Acetyl-3,7-dihydroxyphenoxazine (Biotium), and 100 μM PMSF (Sigma-Aldrich). The real-time formation of fluorescent resorufin (Ex 530 nm/Em 590 nm) was read at 60 s intervals for 10 min at 37°C in a Synergy H1 fluorometer (BioTek). In order to assess the amount of H_2_O_2_ produced by NADPH oxidases, DMSO (vehicle; EMD Millipore) or 10 μM of the NADPH oxidases inhibitor DPI (Cayman Chemical) were added after the first 10 min of kinetic reading. The reaction was then run for another 20 min at 60 s intervals. Immediately after H_2_O_2_ measurement, MTT (ATCC) assay was performed (per manufacturer’s instructions) to normalize H_2_O_2_ production to the amount of viable cells. All samples were assayed in triplicates. The amount of H_2_O_2_ was determined using a standard curve of H_2_O_2_ prepared fresh for each experiment. Data are expressed as the rate of H_2_O_2_ release over the last 8 min for non-DPI experiments or 18 min for experiments using DPI.

### Myeloperoxidase Activity

Snap-frozen longitudinal colon sections were homogenized in 50 mM phosphate buffer containing 13.7 mM of hexadecyltrimethylammonium bromide (Sigma Aldrich) by means of a GentleMACS dissociator (Miltenyi Biotec). MPO activity of the supernatants was determined by measuring their ability to oxidize *o*-dianisidine (Sigma Aldrich) in the presence of H_2_O_2_ and interpolating their values to those of a known MPO standard. The MPO activity was finally normalized to the initial weight of each sample.

### Quantitative PCR Analysis

RNA from colonoids or IECs lysed in Trizol was isolated using phenol-chloroform extraction (Chomczynski and Sacchi, 1987). 50 and 500 ng RNA, respectively, were retrotranscribed using PrimeScript RT reagent Kit (Takara Bio Inc.), and the resulting cDNA was amplified on a LightCycler 480 II instrument (Roche Applied Science) in the presence of selected primers (Supplementary Table 2) using the SYBR Premix Ex Taq (Takara). mRNA expression levels were calculated by means of the ΔΔCt method (Livak and Schmittgen, 2001) and normalized to the geometric mean of the housekeeping genes β-actin and glucuronidase-β.

### Statistical Analysis

Results are presented as mean values and standard deviation (SD). Except for the microbiome data, all of the other data analysis and plots were performed using Prism8 (GraphPad Software, Inc.) and compared using Student’s *t*-test, one-way ANOVA, or two-way ANOVA, as indicated. For the microbiome analysis, MicrobiomeAnalyst was used to perform non-parametric multivariate ANOVA (PEMANOVA) to assess the statistical significance of the principal component analysis (Dhariwal et al., 2017). A *P* value of < 0.05 was considered to be significant. All datasets can be found in Supplementary Data Sheet 1.

## Results

### Accurate Measurement of Epithelial H_2_O_2_ Release Requires the Inhibition of Carboxylesterases

**FIGURE 1:**
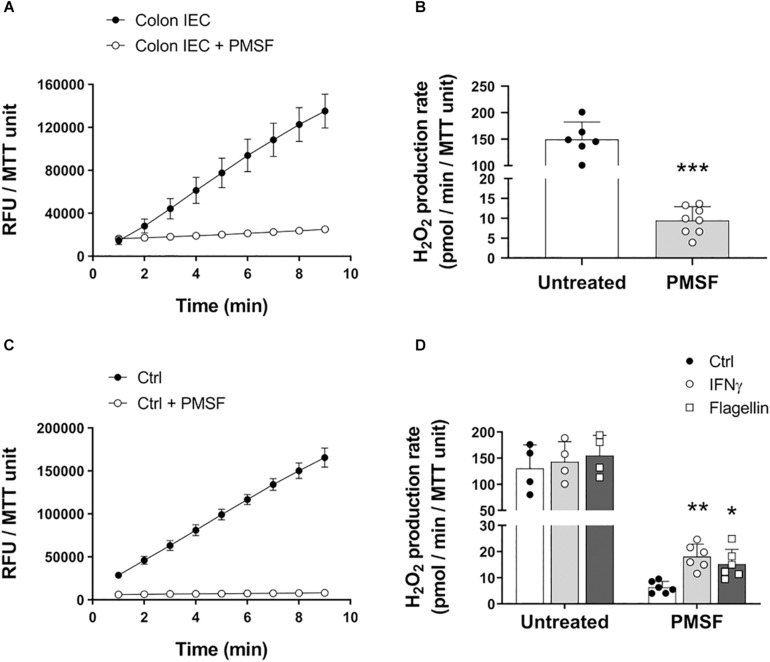
Carboxylesterase activity can obscure measurement of H_2_O_2_ released by IECs. IECs were assayed for their ability to oxidize AR in the presence or absence of PMSF *ex vivo* and *in vitro*. **(A)** Representative experiment showing real-time oxidation kinetics of AR in freshly isolated IECs with or without PMSF. Samples were run in triplicate. RFU, relative fluorescence units. **(B)** AR oxidation rate by freshly isolated IECs in the presence or absence of PMSF. PMSF vs. untreated, ^∗∗∗^*P* < 0.001 as determined by two-tailed Student’s *t*-test (*n* = 6–8 mice). **(C)** Real-time oxidation kinetics of AR in cultured colonoids. **(D)** AR oxidation rate by cultured colonoids in response to inflammatory and microbial stimuli in the presence or absence of PMSF. IFNγ vs. control, ^∗∗^*P* < 0.01; Flagellin vs. control, ^∗^*P* < 0.05 as determined by one-way ANOVA followed by Dunnett’s *post hoc* test (*n* = 6 colonoid cultures). Two-way ANOVA additionally identified a significant overall effect for PMSF, *P* < 0.001 (*n* = 4–6 colonoid cultures).

Cultured cells and tissues, such as liver and kidney, contain carboxylesterases that oxidize AR to resorufin even in the absence of HRP (Miwa et al., 2016). Given that IECs express carboxylesterases (Jones et al., 2013), we first sought to determine whether these enzymes impact the oxidation of AR distorting the measurement of epithelial-released H_2_O_2_. In order to inhibit carboxylesterase-mediated oxidation, freshly isolated IECs from mouse colon were incubated with PMSF and used to determine the formation of resorufin. In the absence of PMSF, colonic IECs induced a high rate of oxidation of AR that was significantly reduced upon addition of PMSF to the reaction buffer (untreated = 149.5 (SD 32.9) vs. PMSF = 9.44 (SD 3.5) pmol/min/MTT unit, *P* < 0.0001; Figures 1A,B), demonstrating that carboxylesterases affect the quantification of H_2_O_2_ production when using this method. Indeed, the high rate of oxidation of AR in the absence of PMSF was in fact HRP-independent, as similar rate values were obtained without the addition of HRP (data not shown). This was also seen in IECs from other sections of the gastrointestinal tract, including the duodenum, jejunum, and ileum, indicating that the distortion of H_2_O_2_ measurements by carboxylesterases is not only limited to the colon (Supplementary Figure 1A). Indeed, we found that inhibition of carboxylesterases revealed significant differences in the production of ROS between duodenum, jejunum, and colon (Supplementary Figure 1A).

To determine whether carboxylesterases also obscure differences in H_2_O_2_ production upon microbial and inflammatory challenge *in vitro*, we treated organoid cultures from mouse colonic IECs (colonoids) with IFNγ and flagellin, and measured oxidation of AR with or without PMSF. Similar to freshly isolated IECs, untreated colonoids caused a high oxidation of AR in the absence of PMSF that was dramatically reduced with the addition of PMSF (Figure 1C). Moreover, inhibition of carboxylesterase activity with PMSF revealed that mouse colonic IECs respond to stimulation with IFNγ and flagellin by releasing H_2_O_2_ to the extracellular milieu (ctrl = 6.3 (SD 2.3) vs. IFNγ = 18.08 (SD 4.8) vs. flagellin = 15.19 (SD 5.6) pmol/min/MTT unit; *P* < 0.01 for IFNγ and *P* < 0.05 for flagellin; Figure 1D). These differences were not observed in colonoids incubated without PMSF (Figure 1D). We additionally characterized the basal production of H_2_O_2_ in human colonoids isolated from biopsies of IBD patients in remission and in human epithelial cell lines. While incubation of PMSF was necessary to quantify the actual production of H_2_O_2_ in steady-state conditions in human colonoids, human IEC lines HT-29 and SW480 did not require neutralization of carboxylesterase activity (Supplementary Figures 1B,C). Human colonoids were also assayed for their responses to IFNγ and flagellin. Whereas we did not observe a consistent increase in H_2_O_2_ upon stimulation with IFNγ, flagellin did cause a higher production of epithelial H_2_O_2_ (ctrl = 7.57 (SD 4.08) vs. flagellin = 11.66 (SD 5.91) pmol/min/MMT unit; *P* < 0.05; Supplementary Figure 1D), demonstrating that human colonoids also respond to microbial stimuli by producing ROS. Taken together, these results suggest that the presence of carboxylesterases in primary IECs masks the actual release of ROS in steady-state and in response to microbial and proinflammatory stimuli.

### Duox2 Mediates Epithelial H_2_O_2_ Production in Response to Proinflammatory and Bacterial Stimuli

**FIGURE 2:**
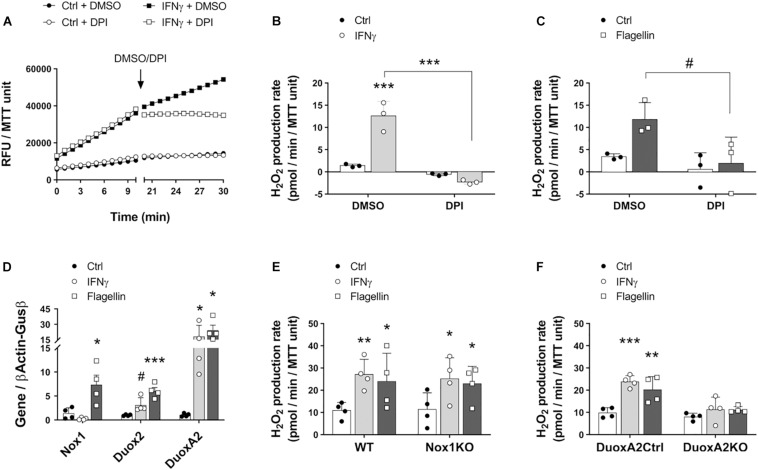
Intestinal epithelial cells in culture respond to inflammatory and microbial stimuli by inducing the synthesis of H_2_O_2_ via Duox2. The involvement of NADPH oxidases Nox1 and Duox2 in epithelial responses to IFNγ and flagellin was determined in cultured colonoids. **(A)** Representative experiment showing real-time production of H_2_O_2_ in response to IFNγ before and after the addition of the NADPH oxidase inhibitor DPI or its vehicle, DMSO. **(B)** H_2_O_2_ production rate in response to IFNγ and in the presence of DPI/DMSO. IFNγ-DMSO vs. IFNγ-DPI, ^∗∗∗^*P* < 0.001 as determined by two-way ANOVA followed by Tukey’s *post hoc* test (*n* = 3 colonoid cultures). **(C)** H_2_O_2_ production rate in response to flagellin and in the presence of DPI/DMSO. Flagellin-DMSO vs. Flagellin-DPI, ^#^*P* = 0.063 as determined by two-way ANOVA followed by Tukey’s *post hoc* test (*n* = 3 colonoid cultures). **(D)** NADPH oxidase expression in cultured IECs stimulated with IFNγ or flagellin. Nox1-flagellin vs. Nox1-control, ^∗^*P* < 0.05; Duox2-IFNγ vs. Duox2-control, ^#^*P* = 0.085; Duox2-flagellin vs. Duox2-control, ^∗∗∗^*P* < 0.001; DuoxA2-IFNγ vs. DuoxA2-control, ^∗^*P* < 0.05; DuoxA2-flagellin vs. DuoxA2-control, ^∗^*P* < 0.05 as determined by two-way ANOVA followed by Tukey’s *post hoc* test (*n* = 4 colonoid cultures). **(E)** Nox1 involvement in IEC responses to IFNγ and flagellin. WT-IFNγ vs. WT-control, ^∗∗^*P* < 0.01; WT-flagellin vs. WT-control, ^∗^*P* < 0.05; Nox1-KO-IFNγ vs. Nox1-KO-control, ^∗^*P* < 0.05; Nox1-KO-flagellin vs. Nox1-KO-control, ^∗^*P* < 0.05 as determined by two-way ANOVA followed by Tukey’s *post hoc* test (*n* = 4 colonoid cultures). **(F)** Duox2 involvement in IEC responses to IFNγ and flagellin. DuoxA2 control-IFNγ vs. DuoxA2 control-control, ^∗∗∗^*P* < 0.001; DuoxA2 control-flagellin vs. DuoxA2 control-control, ^∗∗^*P* < 0.01 as determined by two-way ANOVA followed by Tukey’s *post hoc* test (*n* = 4 colonoid cultures).

Previous work has demonstrated that human airway epithelial cells produce H_2_O_2_ in response to IFNγ and flagellin in a NADPH oxidase-dependent manner (Gattas et al., 2009). Furthermore, increased mucosal abundance of Enterobacteriaceae in the gut has been associated with a higher expression of the NADPH oxidases Duox2 and DuoxA2 (Grasberger et al., 2015). Given that many species of enterobacteria are flagellated, we asked whether the structural component of flagella, flagellin, and IFNγ induced these enzymes in IECs to produce H_2_O_2_ and whether our modified assay could accurately quantify the resulting H_2_O_2_ production. Colonoids in culture were incubated overnight with IFNγ or flagellin, and the conversion of AR to resorufin was measured in the presence of PMSF. To test the dependence of H_2_O_2_ production on NADPH oxidases, we added the inhibitor DPI to colonoid cultures. Our results demonstrate that incubation with IFNγ and flagellin elicited an increase in the production of H_2_O_2_ in colonoids that was totally inhibited by treatment with DPI but not by its vehicle, DMSO (Figures 2A–C). These results suggest that colonoids respond to proinflammatory stimuli and bacterial motifs by producing ROS in a NADPH oxidase-mediated fashion. Furthermore, these results validate the specificity of this modified AR assay in measuring IEC-released H_2_O_2_.

Nox1 and Duox2 are the two main inducible NADPH oxidases in the intestinal epithelium (Grasberger et al., 2015; Aviello and Knaus, 2018). To interrogate whether these enzymes are associated with the redox responses that we observed toward IFNγ and flagellin, we stimulated wild type colonoids with these molecules and analyzed the expression of Nox1, Duox2, and the Duox2 maturation factor DuoxA2. Stimulation with the proinflammatory cytokine IFNγ slightly upregulated Duox2 (ctrl = 1.01 (SD 0.16) vs. IFNγ = 3.12 (SD 0.76) folds, *P* = 0.085) and significantly increased the expression of DuoxA2 transcripts (ctrl = 1.04 (SD 0.34) vs. IFNγ = 18.15 (SD 10.83) folds, *P* < 0.05; Figure 2D). Moreover, the bacterial ligand flagellin significantly upregulated Nox1 (ctrl = 1.4 (SD 1.14) vs. flagellin = 7.34 (SD 2.01) folds, *P* < 0.05), Duox2 (ctrl = 1.01 (SD 0.16) vs. flagellin = 6.04 (SD 1.46) folds, *P* < 0.001) and DuoxA2 (ctrl = 1.04 (SD 0.34) vs. flagellin = 24.13 (SD 10.07) folds, *P* < 0.05; Figure 2D). These findings suggest that both Nox1 and Duox2 might participate in enhancing the release of epithelial H_2_O_2_ upon proinflammatory challenges. To further determine whether Nox1 and Duox2 drive these responses, we prepared colonoids from Nox1-KO mice, epithelial DuoxA2-KO mice and their corresponding wild type littermates and incubated them with IFNγ or flagellin. We observed that Nox1- and Duox2-deficient colonoids displayed a similar basal production of H_2_O_2_ compared to their matched control colonoids. However, while Nox1-KO colonoids responded to IFNγ and flagellin by significantly increasing H_2_O_2_ production (ctrl = 11.41 (SD 7.43) vs. IFNγ = 25.13 (SD 9.49) vs. flagellin = 22.92 (SD 7.85) pmol/min/MTT unit, *P* < 0.05 for both molecules; Figure 2E), DuoxA2-KO colonoids failed to respond to these stimuli (ctrl = 8.08 (SD 1.56) vs. IFNγ = 11.35 (SD 5.49) vs. flagellin = 11.19 (SD 1.45) pmol/min/MTT unit; Figure 2F). These results demonstrate that the increase in the epithelial production of ROS in response to proinflammatory and microbial stimuli is mediated by Duox2.

### Chronic but Not Acute Inflammation Results in Intestinal Epithelial Production of H_2_O_2_

**FIGURE 3:**
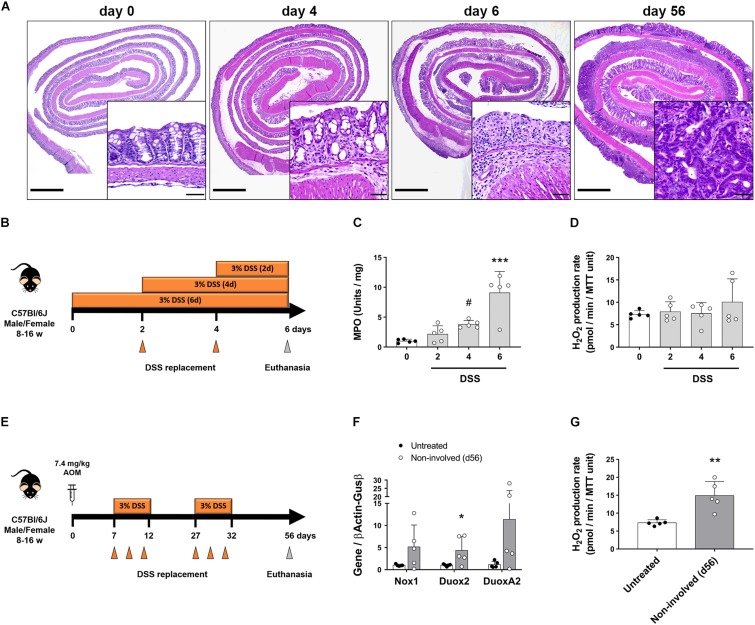
Intestinal epithelial cells upregulate Duox2 and increase their production of H_2_O_2_ during chronic inflammation. The expression of NADPH oxidases and the production of H_2_O_2_ were analyzed in mice undergoing models of acute colitis and tumorigenesis. **(A)** Representative micrographs demonstrating patchy inflammation on day 4 that was extended to most of the colon on day 6. AOM-DSS also induced the formation of dysplastic lesions (56-day inset). Inset scale bar = 50 μm; micrograph scale bar = 1 mm. **(B)** Acute colitis experiments diagram. **(C)** MPO activity in colon of DSS-treated mice. Day4 vs. day0, ^#^*P* = 0.09; day 6 vs. day 0, ^∗∗∗^*P* < 0.001 as determined by one-way ANOVA followed by Dunnett’s *post hoc* test (*n* = 5 mice). **(D)** H_2_O_2_ production rate in IECs isolated from colon of DSS-treated mice. **(E)** Tumorigenesis experiments diagram. **(F)** NADPH oxidase expression in IECs isolated from non-involved areas of AOM-DSS-treated mice on day 56. Duox2-non-involved vs. Duox2-untreated, ^∗^*P* < 0.05 as determined by two-tailed Student’s *t*-test. **(G)** H_2_O_2_ production rate in IECs isolated from non-involved areas of AOM-DSS-treated mice on day 56. Non-involved vs. untreated, ^∗∗^*P* < 0.01 as determined by two-tailed Student’s *t*-test (*n* = 5 mice).

Various studies have implicated Duox2 in intestinal inflammation and as an early signal in VEO-IBD (Haberman et al., 2015; Sommer and Backhed, 2015). However, the functional consequences of Duox2 overexpression has not been addressed. To determine whether increased epithelial-driven H_2_O_2_ production is an early event in intestinal inflammation, we treated mice for various lengths of time with DSS and interrogated the timing of H_2_O_2_ release in response to inflammation (Figure 3B). We confirmed that DSS induced inflammation on days 4 and 6 by histology and using an MPO assay. Histological sections on day 4 were characterized by discrete areas of crypt destruction, edema and immune cell infiltration of the mucosa and submucosa, which became extensive and involved most of the colon by day 6 (Figure 3A). Accordingly, while on day 4 we saw a trend of increased MPO activity, there was a significant increase in MPO on day 6 (day 0 = 1.01 (SD 0.3) vs. day 4 = 3.81 (SD 0.63) vs. day 6 = 9.1 (SD 3.54), *P* = 0.089 and *P* < 0.0001, respectively; Figure 3C). In spite of acute inflammation, we did not find that isolated IECs increased production of H_2_O_2_ (Figure 3D). This was corroborated by a lack of induction of NADPH oxidases at the mRNA level (Supplementary Figures 2A–C). These data demonstrate that the intestinal epithelium does not contribute to an oxidative environment during acute inflammation.

We next asked whether chronic inflammation or neoplasia changed the potential of IECs to release H_2_O_2_. To address this question, we challenged mice with AOM-DSS (Figure 3E) and isolated IECs from specific segments of the mucosa – either from dysplastic tumors or surrounding non-involved (non-dysplastic and non-inflamed) mucosa. Our modified AR assay measured significantly higher levels of H_2_O_2_ in freshly isolated IECs from polypoid tumors compared with surrounding IECs (Supplementary Figure 2D), corroborating previous findings that tumorigenic cells have a higher redox activity than steady-state cells (Schieber and Chandel, 2014). Following AOM-DSS, IECs isolated from non-involved areas expressed significantly higher levels of Duox2 transcripts when compared to IECs in untreated mice (untreated = 1.03 (SD 0.24) vs. non-involved = 4.35 (SD 3.13), *P* < 0.05; Figure 3F). This upregulation in NADPH oxidases translated into a significantly increased production of H_2_O_2_ (untreated = 7.33 (SD 0.83) vs. non-involved = 14.89 (SD 3.97), *P* < 0.01; Figure 3G). Taken together, these data demonstrate that animal models of colitis-associated neoplasia have increased epithelial production of H_2_O_2_ along with increased expression of NADPH oxidases. Even though all the AOM-DSS-treated mice had higher H_2_O_2_ levels in IECs from non-involved areas, MPO activity was not significantly different between non-involved areas of AOM-DSS-treated and untreated mice (Supplementary Figure 2E). These data suggest that IECs produce H_2_O_2_ in the setting of chronic injury even in the absence of inflammation.

### Intestinal Epithelial Cells Upregulate Duox2 and Release H_2_O_2_ in Response to Dysbiosis

**FIGURE 4:**
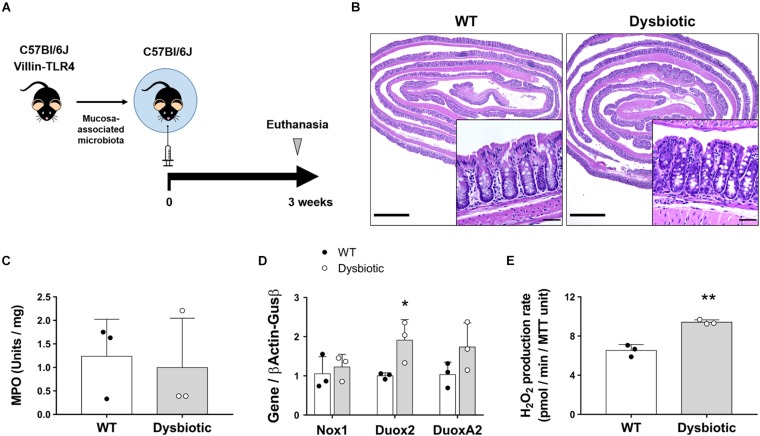
Dysbiosis induces upregulation of Duox2 and enhances the epithelial release of H_2_O_2_. Germ-free mice were colonized with the mucosa-associated microbiota of WT or dysbiotic (villin-TLR4) mice and their epithelial responses were analyzed after a 3-week engraftment. **(A)** Engraftment experiments diagram. **(B)** Representative micrographs showing no signs of inflammation and no major morphological differences between WT- and dysbiotic-microbiota recipient mice. Inset scale bar = 50 μm; micrograph scale bar = 1 mm. **(C)** MPO activity in colon from engrafted mice. **(D)** NADPH oxidase expression in IECs isolated from engrafted mice. Duox2-dysbiotic vs. Duox2-WT, ^∗^*P* < 0.05 as determined by two-tailed Student’s *t*-test (*n* = 3 mice). **(E)** H_2_O_2_ production rate in IECs isolated from engrafted mice. Dysbiotic vs. WT, ^∗∗^*P* < 0.01 as determined by two-tailed Student’s *t*-test (*n* = 3 mice).

Dysbiosis is an indirect consequence of both acute and chronic inflammation and has been shown to upregulate Duox2 (Grasberger et al., 2015; Fritsch and Abreu, 2019). However, whether this translates into a functional increase in the synthesis of ROS by IECs has not been directly demonstrated. We hypothesized that the overexpression of Duox2 during dysbiosis is associated with an increased epithelial H_2_O_2_ production. To investigate dysbiosis as a stimulus for H_2_O_2_, we used dysbiotic mucosa-associated microbiota from our transgenic villin-TLR4 mice (Shang et al., 2008; Dheer et al., 2016). We transferred mucosa-associated microbiota from a pool of WT or villin-TLR4 donor mice to WT GF recipient mice (Figure 4A). After a 3-week engraftment, the microbial populations were analyzed for the stability of engraftment, and the IECs from recipient mice were analyzed for the expression of NADPH oxidases as well as H_2_O_2_ production. 16s rRNA sequencing of donor and recipient mucosa-associated microbiota samples revealed a stable transfer of more than 80% of the phylum, class, order, and genus-level taxa (Supplementary Table 3) that resulted in a similar overall clustering pattern between the donors and their corresponding recipients (Supplementary Figure 3A). The GF mice that received dysbiotic mucosa had a decreased Shannon alpha diversity, an indicator of dysbiosis (Supplementary Figure 3B). Transference of mucosa-associated microbiota did not cause inflammation, as demonstrated by the normal histology of the colon in all recipient mice (Figure 4B) and the low values of MPO activity (Figure 4C).

At a transcriptional level, we found that transfer of mucosa-associated microbiota led to similar expression levels of Nox1 in IECs regardless of donor type. Conversely, the microbiota of the dysbiotic mice elicited an increased expression of Duox2 when compared to WT microbiota in colonic IECs of recipient mice (WT recipient = 1.0 (SD 0.08) vs. dysbiotic recipient = 1.91 (SD 0.53) folds, *P* < 0.05; Figure 4D). Consistent with these findings, IECs isolated from dysbiotic microbiota-recipient mice had a higher production of H_2_O_2_ when compared to WT microbiota-recipient mice (WT recipient = 6.53 (SD 0.6) vs. dysbiotic recipient = 9.42 (SD 0.24) pmol/min/MTT unit, *P* < 0.01; Figure 4E). These findings indicate that, in the absence of inflammation, IECs respond to dysbiosis by upregulating Duox2 and by subsequently inducing the production of H_2_O_2_.

**FIGURE 5:**
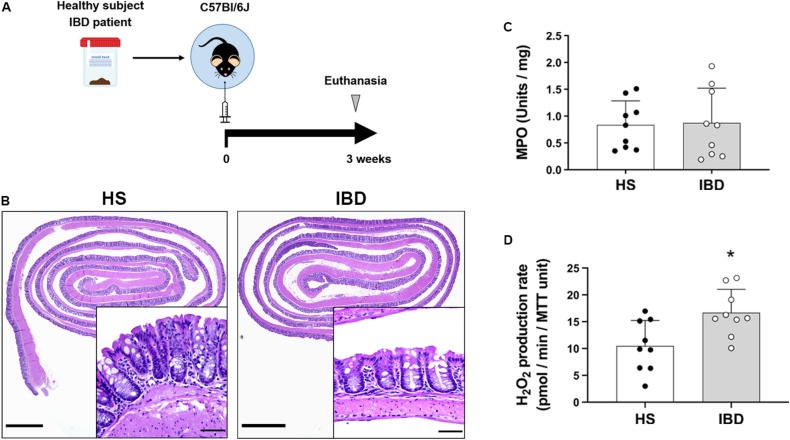
Intestinal epithelial cells increase H_2_O_2_ synthesis in response to the microbiota of IBD patients. The epithelial production of H_2_O_2_ was evaluated in humanized germ-free mice engrafted with the stool microbiota of HS or IBD patients. **(A)** Humanization experiments diagram. **(B)** Representative micrographs showing no signs of inflammation and no major morphological differences between mice engrafted with healthy subjects (HS) or IBD patient stool microbiota. Inset scale bar = 50 μm; micrograph scale bar = 1 mm. **(C)** MPO activity in colon from engrafted mice. **(D)** H_2_O_2_ production rate in IECs isolated from engrafted mice. IBD vs. HS, ^∗^*P* < 0.05 as determined by two-tailed Student’s *t*-test (*n* = 9 mice).

We next asked whether engraftment of GF mice with dysbiotic human microbiota increased epithelial production of H_2_O_2_. We humanized GF mice with stool of either healthy subjects (HS) or IBD patients with no signs of inflammation (Figure 5A) and studied epithelial responses to human dysbiosis. 16s rRNA sequencing revealed that, compared to HS donors, IBD donors had a higher proportion of facultative anaerobes, particularly Proteobacteria and Actinobacteria (Supplementary Figure 4A). Engraftment of human microbiota did not induce inflammation, as determined by histological examination and MPO activity (Figures 5B,C). Previous work has shown that humanization of GF mice with the dysbiotic microbiota from IBD patients induces an increase in Duox2 expression in IECs (Grasberger et al., 2015). We found that IBD dysbiosis induced IECs to increase their production of H_2_O_2_ compared to HS microbiota (HS = 10.49 (SD 4.75) vs. IBD = 16.68 (SD 4.36), *P* < 0.05; Figure 5D). One healthy subject (HS2) with no known disease had dysbiotic microbiota with a decreased proportion of Firmicutes and an increased proportion of facultative anaerobes (Actinobacteria) that was accompanied by a higher, although not significant, production of H_2_O_2_ compared to the other healthy donors (HS1 = 8.15 (SD 6.28) vs. HS2 = 14.63 (SD 2.81) vs. HS3 = 8.68 (SD 1.99); Supplementary Figure 4B). Our data demonstrate that dysbiosis can lead to increased local production of H_2_O_2_ even in the absence of histologic or biochemical inflammation. These data suggest that dysbiosis can be a first step toward abnormal immune activation in the gut and that IECs may play an active role in this process.

## Discussion

To maintain gut homeostasis, IECs recognize microbial motifs and metabolites and respond by producing peptides and other molecules that interact with microbes. One form of bidirectional epithelial-microbial interaction involves the synthesis and release of ROS. However, the short half-life of these molecules and the inadequacy of common methodologies to measure epithelial H_2_O_2_ at low nanomolar ranges hinders the ability to study ROS as signaling intermediates. Here, we took advantage of a modified AR assay to demonstrate that, *in vitro*, inflammatory and microbial stimuli induce the epithelial release of H_2_O_2_ in a Duox2-dependent manner. Furthermore, we report that IECs release H_2_O_2_ into the extracellular milieu in animal models of chronic inflammation and dysbiosis. To our knowledge, this is the first report that shows a direct production of epithelial H_2_O_2_ in response to murine and IBD-driven dysbiosis, supporting the idea that epithelial ROS is a potential mechanism of host-microbe crosstalk in the intestine. Moreover, our results suggest that Duox2 plays a key role in mediating the production of H_2_O_2_ in response to dysbiotic microbiota and chronic inflammation.

Non-phagocytic cells, including IECs, produce ROS at low nanomolar concentrations. The only common method that determines extracellular ROS at the nanomolar level measures the oxidation of AR into resorufin in the presence of HRP and H_2_O_2_ (Dikalov and Harrison, 2014). Even though this method is highly specific and sensitive, it is hindered by the presence of carboxylesterases, which oxidize AR to resorufin in an HRP- and H_2_O_2_-independent manner. While it is known that these esterases distort H_2_O_2_ determination in other cell types from liver and kidney (Miwa et al., 2016), it is not known whether they impact the measurement of H_2_O_2_ in IECs. Our findings demonstrate that modification of the AR assay with PMSF is necessary to quantify H_2_O_2_ released by primary IECs isolated from duodenum, jejunum, ileum, or colon, which are known to express several types of carboxylesterases (Jones et al., 2013). We were able to validate the specificity of H_2_O_2_ release by demonstrating increased production with inflammatory mediators and using a pan-NADPH oxidase inhibitor, DPI, to inhibit epithelial H_2_O_2_ production. In addition, the production of H_2_O_2_ was markedly increased in IECs isolated from tumors, which are known to have high redox activity (Schieber and Chandel, 2014). In contrast to primary IECs, the human IEC lines we tested did not require the inhibition of esterases for the measurement of H_2_O_2_ in steady-state conditions. This observation supports why previous studies were able to determine the H_2_O_2_ release of IEC lines in response to enteric bacteria without modifying the AR assay (Corcionivoschi et al., 2012; MacFie et al., 2014; Alvarez et al., 2016). However, the use of 3D primary cultures is likely to gradually displace cell lines given their increased genetic stability and their functional similarity to normal IECs *in vivo*. Our data strongly support that this modified AR assay provides a valuable tool to precisely quantify the amounts of H_2_O_2_ released by primary IECs, both *ex vivo* and *in vitro*. This method will contribute to improved understanding of the host-microbe interactions mediated through the release of ROS.

IECs are in constant interaction with the microbiota and *lamina propria* cells. Here, we challenged IECs *in vitro* and *in vivo* with stimuli coming from the host (IFNγ/inflammation) and the microbiota (flagellin/dysbiosis) and found a release of epithelial H_2_O_2_ at nanomolar concentrations. At these levels, H_2_O_2_ is likely to participate in host and microbial signaling transduction by altering the phosphotyrosine signaling network and inducing redox-sensitive transcription factors (Kumar et al., 1992; Xanthoudakis and Curran, 1992; Glineur et al., 2000; Leslie et al., 2003; Aviello and Knaus, 2018). For example, in the host, increased levels of H_2_O_2_ have been shown to either enhance inflammation (Chu et al., 2017) or mediate mucosal healing by promoting stem cell proliferation and IEC migration (Buchon et al., 2009; Leoni et al., 2013; Pircalabioru et al., 2016). In bacteria, H_2_O_2_ can have bacteriostatic effects and alter bacterial enzymes involved in the synthesis of virulence factors, reducing the bacterial fitness advantage (Ha et al., 2005; Corcionivoschi et al., 2012; Grasberger et al., 2013; Hayes et al., 2015; Alvarez et al., 2016). Gaining insight into the type of IEC that drives epithelial H_2_O_2_ and its location could potentially provide clarification of the cellular targets and downstream implications of H_2_O_2_. Given that Duox2 is expressed in the tips of the crypt (MacFie et al., 2014; Sommer and Backhed, 2015), we believe that the IECs driving this dysbiosis-induced H_2_O_2_ production are either absorptive enterocytes or goblet cells. These are the most exposed IECs to the microbiota due to their location in the crypt. Conversely, Nox1, which is expressed in the lower two-thirds of the colon crypts, did not play a major role in mediating dysbiosis-induced H_2_O_2_ production (Geiszt et al., 2003; Moll et al., 2018). Therefore, Duox2-mediated ROS is probably more involved in regulating the microbiota opposed to having host inflammatory effects.

In the intestinal epithelium, Duox2 is upregulated by microbial stimuli, including dysbiotic flora and segmented filamentous bacteria (Haberman et al., 2014; Grasberger et al., 2015). Here, we demonstrate that the upregulation of NADPH oxidases in response to inflammatory and microbial stimuli translates into a Duox2-dependent epithelial increase in the production of H_2_O_2_. Indeed, Duox2-deficient colonoids failed to increase H_2_O_2_ synthesis in response to both types of stimuli. Our findings also suggest that the upregulation of Duox2 and subsequent functional increase in H_2_O_2_ requires chronic inflammation or chronic exposure to dysbiotic microbiota. Even though there was apparent inflammation on days 4 and 6, we did not see an increase in epithelial H_2_O_2_ production. By contrast, after chronic inflammation, non-involved areas without inflammation had an increase in the production of H_2_O_2_. Taken together, these observations suggest that there is another driver of Duox2-mediated H_2_O_2_ production in IECs that is not acute inflammation. Our results support that one such driver could be dysbiosis, which is a known inducer of Duox2 expression (Grasberger et al., 2015). Indeed, we saw that not only murine and IBD dysbiosis but also dysbiosis from a HS enhanced the production of epithelial H_2_O_2_. Since time-course studies in the DSS model of colitis have demonstrated that dysbiosis occurs later on day 8, the lack of a significant increase in H_2_O_2_ production during acute inflammation at day 6 would be expected if dysbiosis were the main driver (Schwab et al., 2014).

The importance of maintaining ROS in homeostasis is exemplified by the fact that IBD patients either have loss of function mutations or an upregulation of Duox2 (Haberman et al., 2014; MacFie et al., 2014; Hayes et al., 2015; Levine et al., 2016; Chu et al., 2017; Parlato et al., 2017). The fact that missense mutations in Duox2 in VEO-IBD patients results in an increased invasiveness of bacteria suggests that the upregulation of this enzyme during inflammation is an attempt to contain dysbiosis (Hayes et al., 2015). Since dysbiosis is intimately linked to IBD (Day and Lopez, 2015; Nagao-Kitamoto et al., 2016; Fritsch and Abreu, 2019; Zhu et al., 2019), it is possible that dysbiosis enhances inflammation through the induction of epithelial ROS. Inflammation subsequently exacerbates dysbiosis by providing ROS-generated substrates for facultative anaerobes to bloom generating a feed-forward inflammatory loop. Our results provide the methodology to tease apart epithelial production of H_2_O_2_ and its role in a feed forward inflammatory cycle seen in IBD and IBD-associated neoplasia.

## Data Availability Statement

All datasets generated for this study are included in the article/Supplementary Material.

## Ethics Statement

The studies involving human participants were reviewed and approved by the University of Miami, Leonard Miller M. School of Medicine, Institutional Review Board. The patients/participants provided their written informed consent to participate in this study. The animal study was reviewed and approved by the Institutional Animal Care and Use Committee (IACUC) at the University of Miami.

## Author Contributions

JB, JF, GC, and MA conceived the study, designed the experiments, and wrote the manuscript. JB, JF, and AS performed the experiments. IF and NB bred, genotyped, and maintained the mice colony, as well as managed the germ free facility. JP-K coordinated patient samples and data. JB and JF analyzed the data. All authors have read the manuscript and approved its contents.

## Conflict of Interest

The authors declare that the research was conducted in the absence of any commercial or financial relationships that could be construed as a potential conflict of interest.

## Abbreviations

AOM: azoxymethane
DPI: diphenyleneiodonium
DSS: dextran sodium sulfate
Duox: dual oxidase
GF: germ free
HRP: horseradish peroxidase
HS: healthy subjects
H_2_O_2_: hydrogen peroxide
IBD: inflammatory bowel diseases
IEC: intestinal epithelial cells
IFN γ: interferon- γ
MPO: myeloperoxidase
Nox: NADPH oxidase
PMSF: phenylmethylsulfonyl fluoride
ROS: reactive oxygen species
VEO-IBD: very early onset IBD
WT: wild-type
