# Supplementary material for: Intestinal Epithelial Cells Respond to Chronic Inflammation and Dysbiosis by Synthesizing H2O2
Source: Front Physiol. 2019 Dec 12;10:1484. doi: 10.3389/fphys.2019.01484 (PMC6921703; doi:10.3389/fphys.2019.01484)
Supplement: Supplementary file 1 [file Image_1.pdf]

Supplementary Material

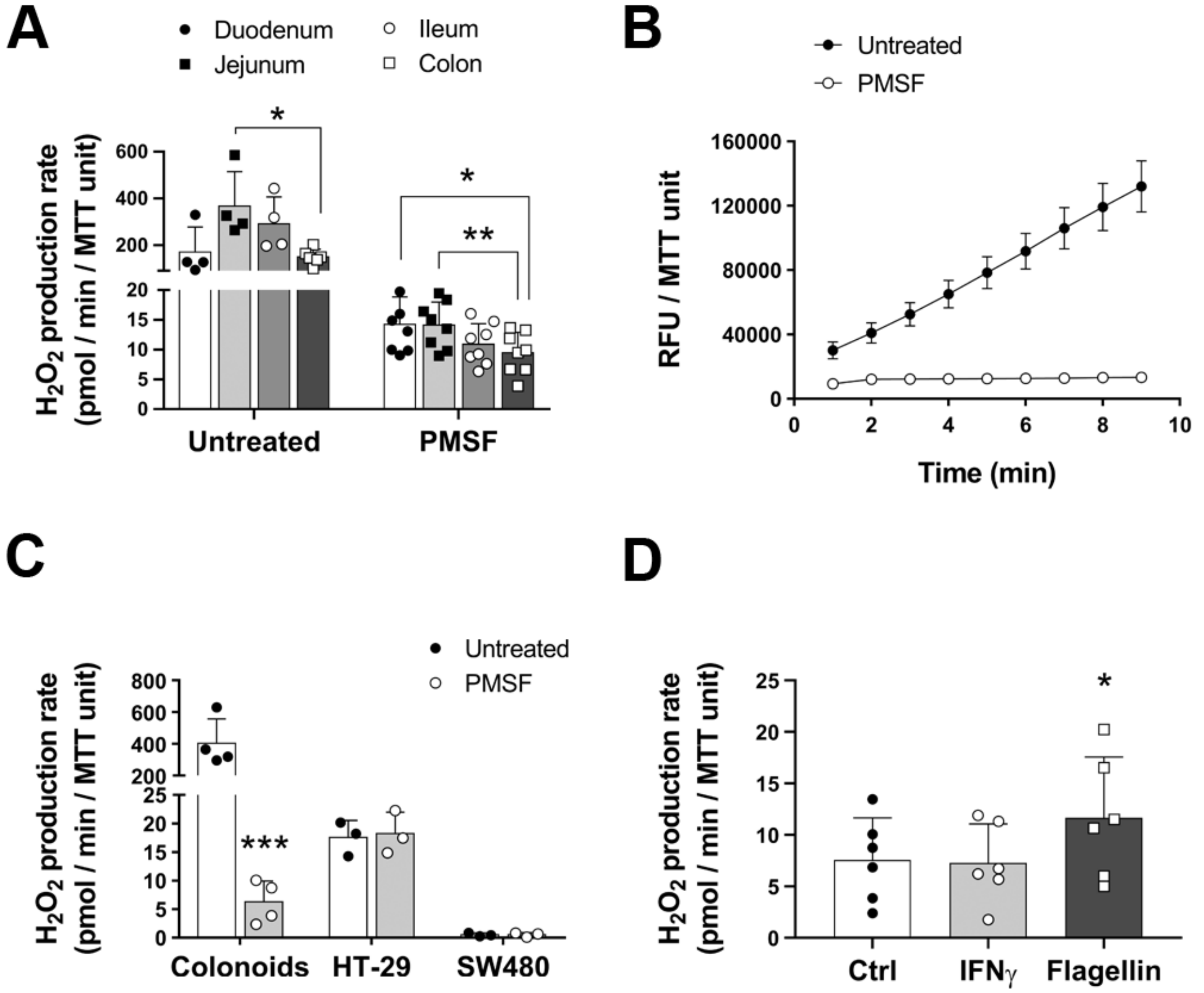

Supplemental Figure 1. Inhibition of carboxylesterases exposes epithelial H_2O_2 production by murine and human IECs. Epithelial production of H_2O_2 was determined in IECs isolated from the different segments of the murine gut or in cultures of human primary IECs. **A)** AR oxidation rate in mouse IECs from duodenum, jejunum, ileum, and colon in the presence or absence of PMSF. Duodenum vs colon, *$P<0.05$; jejunum vs colon, **$P<0.01$ as determined by one-way ANOVA followed by Tukey's post-hoc test (n=8 mice). Two-way ANOVA additionally identified PMSF treatment as a significant source of variability ($P<0.001$). **B)** Representative experiment showing real-time oxidation kinetics of AR in cultured human colonoids in the presence or absence of PMSF. **C)** AR oxidation rate in human colonoids and the human IEC lines HT-29 and SW480 in the presence or absence of PMSF. Colonoids-PMSF vs colonoids-untreated, ***$P<0.001$ as determined by two-way ANOVA followed by Sidak's post-hoc test (n=3-4 cultures). **D)** H_2O_2 production rate in human colonoids in response to IFNγ and flagellin. Flagellin vs control, $P<0.05$ as determined by one-way ANOVA with matched data analysis followed by Dunnett's post-hoc test (n=6 colonoid cultures).

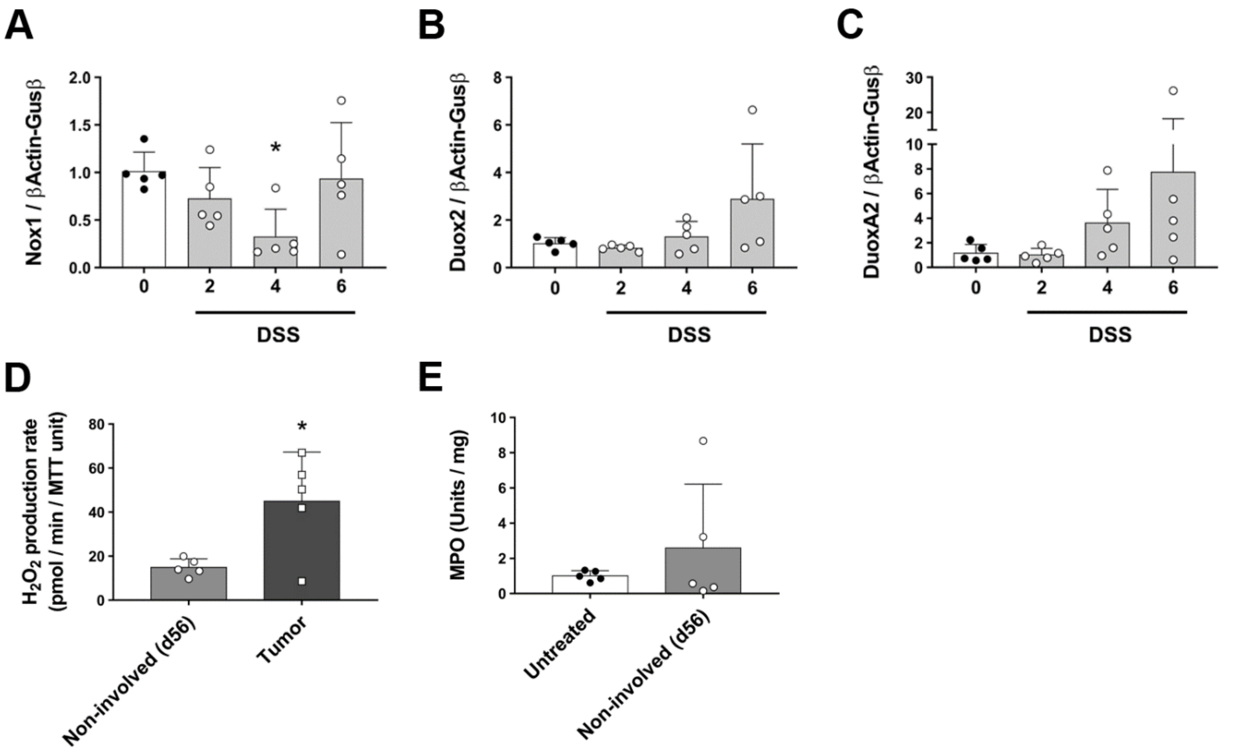

Supplemental Figure 2. Acute inflammation does not consistently upregulate NADPH oxidases. The expression of NADPH oxidases Nox1 **(A)**, Duox2 **(B)**, and DuoxA2 **(C)** in mouse IEC was determined during acute DSS colitis. Nox1 day4 vs day0, **P*<0.05 as determined by one-way ANOVA followed by Dunnett's post-hoc test (n=5 mice). In addition, isolated IECs and their matched colon sections from mice undergoing the AOM-DSS model were analyzed for their production of H_2O_2. **D)** H_2O_2 production rate in IECs isolated from tumors and non-involved areas. Tumor vs non-involved, **P*<0.05 as determined by two-tailed Student's t-test (n=5 mice). **E)** MPO activity in colon sections of untreated mice and non-involved areas on day 56 of AOM-DSS model.

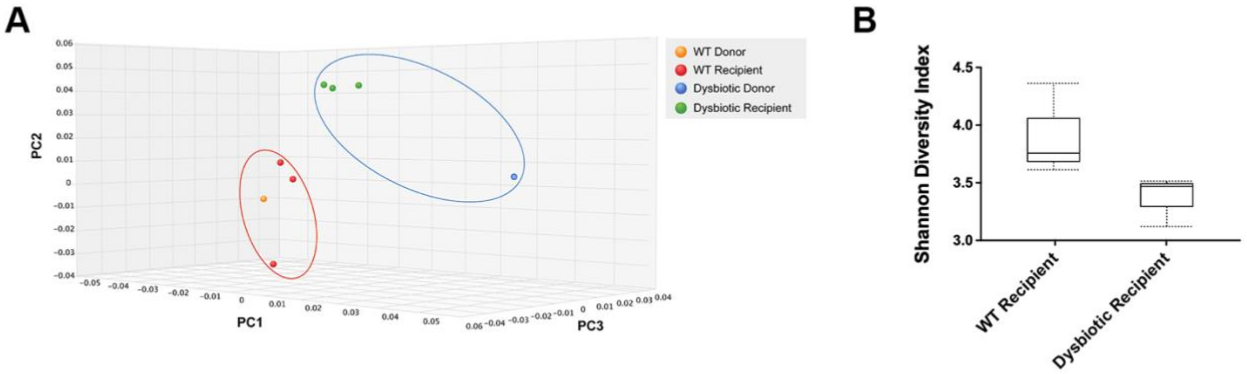

Supplemental Figure 3. Transfer of mucosa-associated microbiota from dysbiotic donor mice leads to dysbiosis in recipient mice. Following engraftment of mucosa-associated microbiota, 16S rRNA gene surveys from both donor and recipient mice were analyzed to validate the engraftment and confirm dysbiosis. **A)** Principal coordinate analysis of WT and dysbiotic donors and their corresponding recipients at the genus level. Principal coordinate 1 (PC1), PC2, and PC3 are scaled on the basis of percent variance. **B)** Shannon diversity indices of bacterial communities in recipient mice (n=3 mice).

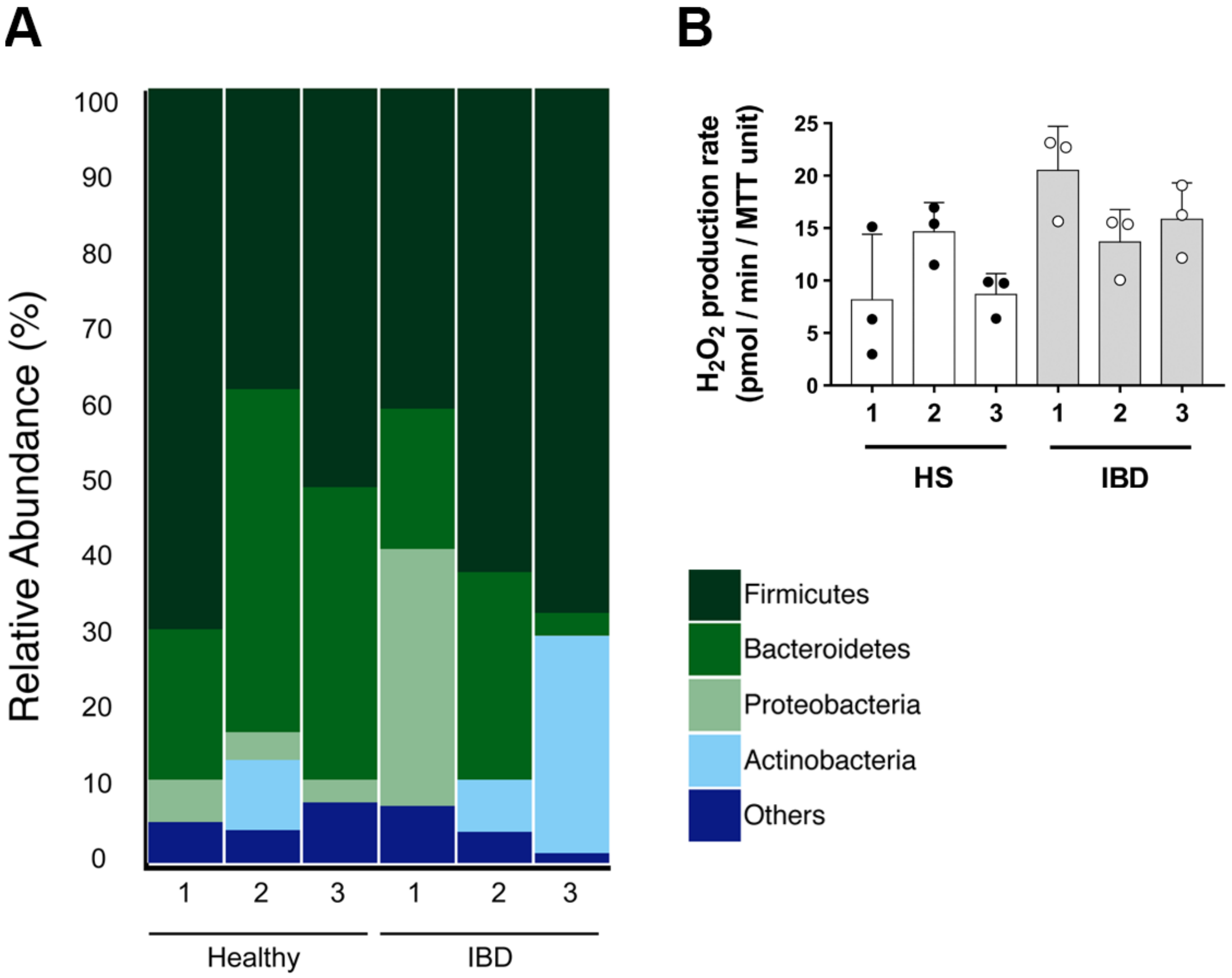

Supplemental Figure 4. Higher abundance of facultative anaerobes in human donor microbiota elicits increased epithelial production of H_2O_2 in recipient germ free mice. The epithelial production of H_2O_2 was evaluated in humanized germ-free mice engrafted with the stool microbiota of healthy subjects or IBD patients. Results are segregated by human donor. **A)** Taxonomic distribution (phylum level taxa) of the stool from healthy subjects and IBD patients. Values represent the average relative abundance from each sample. **B)** H_2O_2 production rate in IECs isolated from engrafted mice (n=3 mice per subject).
