# Supplementary material for: Intestinal Epithelial Cells Respond to Chronic Inflammation and Dysbiosis by Synthesizing H2O2
Source: Front Physiol. 2019 Dec 12;10:1484. doi: 10.3389/fphys.2019.01484 (PMC6921703; doi:10.3389/fphys.2019.01484)
Supplement: Supplementary file 2 [file Table_1.docx]

**Supplemental Table 1: Patient Information**

| **ID** | **Gender** | **Ethnicity** | **Diagnosis** | **Tissue** | **Disease State** |
| --- | --- | --- | --- | --- | --- |
| Organoid 1 | Male | Non-Hispanic | UC | Ascending colon | Non-inflamed |
| Organoid 2 | Male | Non-Hispanic | UC | Sigmoid colon | Non-inflamed |
| Organoid 3 | Female | Hispanic | CD | Ascending colon | Non-inflamed |
| Organoid 4 | Male | Hispanic | CD | Ascending colon | Non-inflamed |
| Organoid 5 | Male | Hispanic | CD | Sigmoid colon | Non-inflamed |
| Organoid 6 | Female | Non-Hispanic | UC | Ascending colon | Non-inflamed |
| HS1 | Male | Non-Hispanic | Healthy | Stool | Non-inflamed |
| HS2 | Male | Hispanic | Healthy | Stool | Non-inflamed |
| HS3 | Female | Non-Hispanic | Healthy | Stool | Non-inflamed |
| IBD1 | Male | Hispanic | UC | Stool | Mild UC, remission |
| IBD2 | Male | Hispanic | UC | Stool | Mild UC, remission |
| IBD3 | Female | Hispanic | UC | Stool | Mild UC, remission |
|  |  |  |  |  |  |

**Supplemental Table 2: qPCR Primers**

| **Gene** | **Sense primer** | **Antisense primer** | **Reference** |  |  |
| --- | --- | --- | --- | --- | --- |
| Gusβ | CCGATTATCCAGAGCGAGTATG | CTCAGCGGTGACTGGTTCG | (Wang et al., 2010) |  |  |
| β-Actin | TGACAGGATGCAGAAGGAGA | CGCTCAGGAGGAGCAATG | (Bamias et al., 2007) |  |  |
| Nox1 | CTGACAAGTACTATTACACGAGAG | CATATATGCCACCAGCTTATGGAAG |  |  |  |
| DuoxA2 | GCCTGGCTTTGCTCACCA | GAGGAGGAGGCTCAGGAT | (Grasberger et al., 2015) |  |  |
| Duox2 | TCCAGAAGGCGCTGAACAG | GCGACCAAAGTGGGTGATG |  |  |  |
|  |  |  |  |  |  |
|  |  |  |  |  |  |

**Supplemental Table 3: Percentage Engraftment**

| **Phylum** | **WT Donor Mean** | **WT GF Mean** | **Std Deviation** | **Dysbiotic Donor Mean** | **Dysbiotic GF Mean** | **Std Deviation** |
| --- | --- | --- | --- | --- | --- | --- |
| Tenericutes | 2.15% | 0.01% | 0.0001 | - | - | - |
| Bacteroidetes | 26.92% | 33.31% | 0.0615 | 32.41% | 39.23% | 0.1094 |
| Cyanobacteria | 5.13% | 4.68% | 0.0758 | 1.80% | 0.32% | 0.0043 |
| Firmicutes | 58.70% | 43.23% | 0.0758 | 44.74% | 22.79% | 0.0295 |
| Actinobacteria | 1.65% | 2.38% | 0.0062 | 6.17% | 6.42% | 0.0189 |
| Verrucomicrobia | 4.50% | 15.46% | 0.1159 | 1.20% | 31.11% | 0.1082 |
| Epsilonbacteraeota | - | - | - | 4.99% | 0.00% | 0.0000 |
| Proteobacteria | - | - | - | 2.85% | 0.05% | 0.0002 |
| Unknown | - | - | - | 4.80% | 0.06% | 0.0001 |
| **Percent Engraftment** | 100% | | | 88% | | |
|  |  |  |  |  |  |  |
| **Class** | **WT Donor Mean** | **WT GF Mean** | **Std Deviation** | **Dysbiotic Donor Mean** | **Dysbiotic GF Mean** | **Std Deviation** |
| Campylobacteria | - | - | - | 4.99% | 0% | 0.0000 |
| Oxyphotobacteria | 5.12% | 4.68% | 0.0757 | 1.67% | 0.32% | 0.0043 |
| Bacilli | 13.18% | 8.46% | 0.0543 | 13.52% | 2.84% | 0.0031 |
| Coriobacteriia | 1.03% | 1.15% | 0.0023 | 1.88% | 5.13% | 0.0217 |
| Unspecified Bacteria | - | - | - | 4.80% | 0.06% | 0.0001 |
| Gammaproteobacteria | - | - | - | 2.59% | 0.02% | 0.0002 |
| Bacteroidia | 26.90% | 33.30% | 0.0614 | 32.39% | 39.19% | 0.1092 |
| Erysipelotrichia | 2.17% | 1.64% | 0.0050 | 10.29% | 0.18% | 0.0021 |
| Verrucomicrobiae | 4.50% | 15.46% | 0.1158 | 1.20% | 31.10% | 0.1082 |
| Mollicutes | 2.15% | 0.01% | 0.0001 | - | - | - |
| Actinobacteria | - | - | - | 4.28% | 1.28% | 0.0030 |
| Clostridia | 43.36% | 33.12% | 0.0751 | 20.90% | 19.77% | 0.0292 |
| **Percent Engraftment** | 100% | | | 91% | | |
|  |  |  |  |  |  |  |
| **Order** | **WT Donor Mean** | **WT GF Mean** | **Std Deviation** | **Dysbiotic Donor Mean** | **Dysbiotic GF Mean** | **Std Deviation** |
| Campylobacterales | - | - | - | 4.99% | 0% | 0.0000 |
| Oxyphotobacteria | 5.11% | 4.67% | 0.0756 | 1.67% | 0.32% | 0.0043 |
| Enterobacteriales | - | - | - | 2.43% | 0% | 0.0000 |
| Lactobacillales | 13.12% | 7.54% | 0.0579 | 13.46% | 2.80% | 0.0034 |
| Bacteroidales | 26.90% | 33.30% | 0.0614 | 32.34% | 39.18% | 0.1091 |
| Bifidobacteriales | 0.56% | 0.70% | 0.0048 | 3.92% | 1.21% | 0.0027 |
| Erysipelotrichales | 2.17% | 1.64% | 0.0050 | 10.29% | 0.18% | 0.0021 |
| Verrucomicrobiales | 4.50% | 15.45% | 0.1158 | 1.20% | 31.10% | 0.1082 |
| Unspecified Bacteria | - | - | - | 4.80% | 0.06% | 0.0001 |
| Coriobacteriales | 1.03% | 1.15% | 0.0023 | 1.88% | 5.13% | 0.0217 |
| Clostridiales | 43.36% | 33.12% | 0.0751 | 20.90% | 19.77% | 0.0292 |
| **Percent Engraftment** | 100% | | | 82% | | |
|  |  |  |  |  |  |  |
| **Family** | **WT Donor Mean** | **WT GF Mean** | **Std Deviation** | **Dysbiotic Donor Mean** | **Dysbiotic GF Mean** | **Std Deviation** |
| Bacteroidales s24-7 family | 6.34% | 8.08% | 0.0127 | 18.69% | 13.08% | 0.0587 |
| Oxyphotobacteria | 5.11% | 4.67% | 0.0756 | 1.67% | 0.32% | 0.0043 |
| Atopobiaceae | - | - | - | 0.55% | 0.65% | 0.0006 |
| Lachnospiraceae | 33.03% | 28.88% | 0.0715 | 15.75% | 18.64% | 0.0308 |
| Helicobacteraceae | - | - | - | 4.99% | 0% | 0.0000 |
| Unspecified Bacteria | - | - | - | 4.80% | 0.06% | 0.0001 |
| Enterobacteriaceae | - | - | - | 2.43% | 0% | 0.0000 |
| Saccharibacteria Unspecified Class | - | - | - | 0.55% | 0% | 0.0000 |
| Anaeroplasmataceae | 1.64% | 0.002% | 0.0000 | - | - | 0.0000 |
| Bacteroidales | 9.35% | 12.23% | 0.0185 | 5.20% | 18.35% | 0.0411 |
| Enterococcaceae | - | - | - | 1.98% | 0.58% | 0.0014 |
| Marinifilaceae | - | - | - | 1.04% | 0% | 0.0000 |
| Clostridiales | 0.83% | 0.01% | 0.0001 | 0.76% | 0% | 0.0000 |
| Lactobacillaceae | 13.05% | 6.41% | 0.0519 | 10.84% | 1.88% | 0.0055 |
| Rikenellaceae | 8.88% | 2.70% | 0.0166 | 4.97% | 6.61% | 0.0262 |
| Ruminococcaceae | 8.84% | 3.20% | 0.0083 | 3.47% | 1.00% | 0.0032 |
| Bifidobacteriaceae | 0.56% | 0.70% | 0.0048 | 3.92% | 1.21% | 0.0027 |
| Coriobacteriaceae | - | - | - | 1.01% | 0.10% | 0.0005 |
| Erysipelotrichaceae | 2.17% | 1.64% | 0.0050 | 10.29% | 0.18% | 0.0021 |
| Bacteroidaceae | 2.03% | 9.85% | 0.0176 | 1.05% | 0.18% | 0.0024 |
| Verrucomicrobiaceae | 4.50% | 15.45% | 0.1158 | 1.20% | 31.09% | 0.1082 |
| Eggerthellaceae | 1.02% | 1.08% | 0.0025 | - | - | - |
| **Percent Engraftment** | 100% | | | 75% | | |
|  |  |  |  |  |  |  |
| **Genus** | **WT Donor Mean** | **WT GF Mean** | **Std Deviation** | **Dysbiotic Donor Mean** | **Dysbiotic GF Mean** | **Std Deviation** |
| Lachnoclostridium | 1.23% | 1.07% | 0.0077 | 1.15% | 2.09% | 0.0116 |
| Clostridiales | 0.83% | 0.68% | 0.0001 | 0.76% | 0% | 0.0000 |
| Odoribacter | - | - | - | 1.04% | 0% | 0.0000 |
| Helicobacter | - | - | - | 4.75% | 0% | 0.0000 |
| Bacteroides | 2.03% | 9.85% | 0.0176 | 1.05% | 0.18% | 0.0024 |
| Adlercreutzia | 1.02% | 1.08% | 0.0025 | - | - | - |
| Unspecified Bacteria | - | - | - | 4.79% | 0.06% | 0.0001 |
| Atopobiaceae | - | - | - | 0.55% | 0.65% | 0.0006 |
| Lachnospiraceae | 20.31% | 17.10% | 0.0131 | 7.20% | 3.90% | 0.0072 |
| Oscillospira | 4.66% | 0.52% | 0.0033 | 1.32% | 0.20% | 0.0035 |
| Collinsella | - | - | - | 0.87% | 0.88% | 0.0000 |
| Coprococcus | 1.25% | 1.09% | 0.0058 | - | - | - |
| Allobaculum | - | - | - | 9.31% | 0.40% | 0.0001 |
| Lactobacillus | 13.03% | 6.41% | 0.0519 | 10.84% | 1.87% | 0.0055 |
| Anaeroplasma | 1.64% | 0.24% | 0.0000 | - | - | - |
| Bacteroidales | 9.35% | 12.23% | 0.0185 | 5.20% | 18.35% | 0.0411 |
| Escherichia-shigella | - | - | - | 1.96% | 0% | 0.0000 |
| Ruminococcaceae | 0.87% | 0.04% | 0.0002 | 1.06% | 0.71% | 0.0047 |
| Blautia | 1.57% | 7.19% | 0.0415 | 0.52% | 6.94% | 0.0182 |
| Oxyphotobacteria | 5.11% | 4.67% | 0.0756 | 1.67% | 0.32% | 0.0043 |
| Candidatus | - | - | - | 0.55% | 0% | 0.0000 |
| Lachnospiraceae nk4a | 6.05% | 1.21% | 0.0017 | 4.14% | 3.43% | 0.0096 |
| Ruminococcus | 1.84% | 2.56% | 0.0054 | - | - | - |
| Rikenellaceae | 8.76% | 2.63% | 0.0160 | 3.93% | 6.45% | 0.0256 |
| Dorea | - | - | - | 1.23% | 0.02% | 0.0001 |
| Bacteroidales s24-7 group | 6.34% | 8.08% | 0.0127 | 18.69% | 13.08% | 0.0587 |
| Bifidobacterium | 0.56% | 0.70% | 0.0048 | 3.91% | 1.21% | 0.0027 |
| Turicibacter | 1.92% | 1.55% | 0.0049 | 0.64% | 0.16% | 0.0022 |
| Enterococcus | - | - | - | 1.96% | 0.58% | 0.0014 |
| Alistipes | - | - | - | 0.93% | 0.12% | 0.0005 |
| Akkermansia | 4.48% | 15.40% | 0.1153 | 1.20% | 31.00% | 0.1078 |
| **Percent Engraftment** | 100% | | | 81% | | |

BAMIAS, G., OKAZAWA, A., RIVERA-NIEVES, J., ARSENEAU, K. O., DE LA RUE, S. A., PIZARRO, T. T. & COMINELLI, F. 2007. Commensal bacteria exacerbate intestinal inflammation but are not essential for the development of murine ileitis. *J Immunol,* 178**,** 1809-18.

GRASBERGER, H., GAO, J., NAGAO-KITAMOTO, H., KITAMOTO, S., ZHANG, M., KAMADA, N., EATON, K. A., EL-ZAATARI, M., SHREINER, A. B., MERCHANT, J. L., OWYANG, C. & KAO, J. Y. 2015. Increased Expression of DUOX2 Is an Epithelial Response to Mucosal Dysbiosis Required for Immune Homeostasis in Mouse Intestine. *Gastroenterology,* 149**,** 1849-59.

WANG, F., WANG, J., LIU, D. & SU, Y. 2010. Normalizing genes for real-time polymerase chain reaction in epithelial and nonepithelial cells of mouse small intestine. *Anal Biochem,* 399**,** 211-7.
